# Characterization of a Bioflocculant Produced by a Consortium of *Halomonas* sp. Okoh and *Micrococcus* sp. Leo

**DOI:** 10.3390/ijerph10105097

**Published:** 2013-10-16

**Authors:** Kunle Okaiyeto, Uchechukwu U. Nwodo, Leonard V. Mabinya, Anthony I. Okoh

**Affiliations:** Applied and Environmental Microbiology Research Group (AEMREG), Department of Biochemistry and Microbiology, University of Fort Hare, Alice 5700, South Africa; E-Mails: UNwodo@ufh.ac.za (U.U.N.); LMabinya@ufh.ac.za (L.V.M.); AOkoh@ufh.ac.za (A.I.O.)

**Keywords:** bioflocculant, flocculating activity, consortium, *Halomonas* sp. Okoh, *Micrococcus* sp. Leo

## Abstract

The physicochemical and flocculating properties of a bioflocculant produced by a bacterial consortium composed of *Halomonas* sp. Okoh and *Micrococcus* sp. Leo were investigated. The purified bioflocculant was cation and pH dependent, and optimally flocculated kaolin clay suspension at a dosage of 0.1 mg/mL. The flocculating activity of the bioflocculant was stimulated in the presence of Ca^2+^, Mn^2+^, Al^3+^ and had a wide pH range of 2–10, with the highest flocculating activity of 86% at pH 8. The bioflocculant was thermostable and retained more than 70% of its flocculating activity after being heated at 80 °C for 30 min. Thermogravimetric analyses revealed a partial thermal decomposition of the biofloculant at 400 °C. The infrared spectrum showed the presence of hydroxyl, carboxyl and amino moieties as functional groups. The bioflocculant produced by the bacterial consortium appears to hold promising alternative to inorganic and synthetic organic flocculants that are widely used in wastewater treatment.

## 1. Introduction

Flocculants are widely used in various industrial processes such as drinking water treatment, downstream processes, wastewater treatment plants, and in different fermentation processes [1]. Although chemical flocculants have numerous advantages of being effective in terms of flocculating efficiency, affordability and availability, their usage has been reported to be harmful to humans [2]. In addition, they have been reported to be neurotoxic, carcinogenic and recalcitrant to degradation, thus constituting environmental nuisances [3]. Due to the adverse nature of chemical synthetic flocculants, more attention has been given to the use of flocculants produced by microorganisms [4].

Xia *et al.* [5] reported that the biopolymers produced by microorganisms during their growth are harmless, degradable, and do not lead to secondary pollution. As a result, bioflocculants are being considered as a good replacement for chemical flocculants used in wastewater treatment and other industrial processes [6]. 

In recent years, many studies have been undertaken where different microorganisms such as algae, fungi, bacteria and actinomycetes have been used in bioflocculant production [5,7]. Although many bioflocculants have been produced by different microbes, low flocculating activity and high production costs have been limiting factors hindering large-scale production [8]. 

According to the findings of Xia *et al.* [5], screening for microorganisms with high bioflocculant-producing capability and high flocculating efficiency is vital for success in this field. Furthermore, Fujita *et al.* [9] investigated the use of low-cost substrates in growth media for bioflocculant production as a possible cost-cutting measure. Some low-cost substrates such as soybean juice, fishmeal wastewater have been documented as alternative nitrogen source components in production media [10,11]. 

The idea of using two or more microbes in consortium for bioflocculant production was first reported by Ma *et al.* [12]. The concept behind this approach was to improve the yield and flocculating efficiency of the bioflocculant produced by individual strains. Furthermore, Zhu *et al.* [13] reported that a compound bioflocculant produced by a mixed culture of strains *Rhizobium radiobacter* F2 and *Bacillus shaeicus* F6 had higher flocculating activity when compared to individual strains. In addition, Wang *et al.* [14] reported that the compound bioflocculant produced by a mixed culture of *Rhizobium radiobacter* F2 and *Bacillus shaeicus* F6 possessed higher flocculating efficiency than those from individual strains.

There is a dearth of information on bioflocculant production by bacterial mixed culture hence, *Halomonas* sp. Okoh and *Micrococcus* sp. Leo isolated from sediment samples of Algoa Bay of the Eastern Cape Province of South Africa, which in our previous studies had demonstrated good potential for the production of bioflocculants as axenic cultures, were investigated as a mixed culture for enhanced bioflocculant yields and the bioflocculant was characterized. 

## 2. Experimental Section

### 2.1. Source of Bacteria

The bacteria was isolated from the sediment of Algoa Bay in the Eastern Cape Province of South Africa and maintained in 20% glycerol at −80 °C as part of the culture collections of the Applied and Environmental Microbiology Research Group (AEMREG), University of Fort Hare, Alice, South Africa. 

### 2.2. Growth Media

The growth medium for bioflocculant production was composed of glucose (20 g), MgSO_4_·7H_2_O (0.2 g), (NH_4_)_2_SO_4_ (0.2 g), K_2_HPO_4_ (5 g), urea (0.5 g), yeast extract (0.5 g) and KH_2_PO_4_ (2 g) in a litre of filtered seawater at pH 6.5 and sterilized by autoclaving at 121–124 °C for 15 min [15]. 

### 2.3. Evaluation of Bioflocculant Production

The bacteria were inoculated into a 250 mL flask containing 50 mL of production medium prepared according to description of Zhang *et al.* [15] and incubated at 28 °C in a shaker at 160 rpm for 5 days and centrifuged at 4,000 × g for 30 min at 4 °C. The cell free culture supernatants were used to determine flocculating activities of the bioflocculant produced by the consortium. 

### 2.4. Determination of Flocculating Activity

Using the description of Kurane *et al.* [16] with minor modifications, kaolin clay was used as the test material for determining the flocculating activity of the produced bioflocculant. Four grams of kaolin clay was suspended in 1 L of distilled water to make a concentration (4 g/L). One hundred millilitres of kaolin suspension was measured into 250 mL flask, 3 mL of 1% w/v CaCl_2_ and added 2 mL of culture supernatant were added. The mixture was agitated vigorously for 60 s and then poured into 100 mL measuring cylinder and allowed to sediment for 5 min at room temperature. The optical density (OD) of the clarifying supernatant was measured at 550 nm with a UV spectrophotometer (Thermo Spectronic, USA) and the flocculating activity determined as follows:

[(A − B/A)] × 100%

where A and B are optical densities of control and sample measured at 550 nm respectively.

### 2.5. Time Course Assay for Bioflocculant Production

The optimum culture conditions and cultivation conditions previously described for the individual strains were used. The strains were pre-cultured in 50 mL growth medium contained in 250 mL flask on the rotary shaker (160 rpm) at 28 °C for inoculation preparation. After 18 h of cultivation, 2% (v/v) culture broth of *Halomonas* sp. Okoh and 4% (v/v) culture broth of *Micrococcus* sp. Leo were inoculated into 200 mL of the production medium in 500 mL flask [14]. Batch fermentation was carried out under the same cultivation conditions as those for pre-cultivation. Medium samples (15 mL) were withdrawn at 12 h intervals and monitored for pH, cell growth, cell count and flocculating activity. Two millilitres of culture broth was centrifuged at 4,000 × g, at 4 °C for 30 min, and the cell free supernatant was used as the test bioflocculant to determine the flocculating activity. The bacterial growth was monitored by measuring the optical density (OD_600_) and bacterial counts was determined by standard spread plate technique using nutrient agar and all plates were incubated at 35 °C for 36 h. 

### 2.6. Extraction and Purification of the Bioflocculant

After 5 days of fermentation, the culture broth was centrifuged at 4,000 × g at 4 °C for 30 min. In order to remove the insoluble substances, one volume of distilled water was added to the supernatant phase and then centrifuged at 4,000 × g for 15 min, 4 °C. Two volumes of ethanol were added to the supernatant, and the solution was agitated then left standing at 4 °C for 12 h. The precipitate was vacuum-dried and then re-dissolved in distilled water (1% w/v) and one volume of a mixture of chloroform and *n*-butyl alcohol (5:2 v/v*)* was added. After agitating, the mixture was left standing at room temperature for 12 h. The supernatant was then be centrifuged at 4,000 × g for 15 min at 4 °C and dialyzed overnight against distilled water overnight. The dialysate was then vacuum-dried in order to obtain a purified bioflocculant. 

### 2.7. Determination of Bioflocculant Dosage (Jar Test)

Different concentrations of the bioflocculant solution (0.02, 0.04, 0.06, 0.08, 0.1, 0.2, 0.3, 0.4 and 0.5 mg/mL) were prepared and their flocculating activities evaluated. Four grams of kaolin clay was weighed and dissolved in 1 L of distilled water. Three millilitres of 1% (w/v) CaCl_2_ and 2 mL of bioflocculant solution were both added to 100 mL kaolin suspension in 500 mL beakers. The solution was agitated at 200 rpm for 3 min and the speed reduced to 45 rpm for further 10 min of agitation [17]. The solution was poured into 100 mL measuring cylinder, allowed to sediment for 10 min and 2 mL of clear supernatant withdrawn and the flocculating activity was read at 550 nm. 

### 2.8. Effect of Cations on Flocculating Activity of Purified Bioflocculant

A solution of bioflocculant concentration 0.1 mg/mL was prepared. The effects of the following salt solutions at 1% w/v NaCl, KCl, LiCl_2_, MgCl_2_, MnCl_2_·4H_2_O, BaCl_2,_ AlCl_3_ and FeCl_3_·6H_2_O on flocculating activity of the purified bioflocculant were determined according to Kurane *et al.* [16].

### 2.9. Effect of pH on Flocculating Activity of Purified Biofloculant

Concentration of 0.1 mg/mL solution of bioflocculant solution was prepared. The adjusted pH of individual kaolin solutions in separate flasks ranged from 2–12 prior to determining flocculating activity at each of these pH values [18]. 

### 2.10. Effect of Temperature on Flocculating Activity of Purified Bioflocculant

The purified bioflocculant was dissolved in distilled water to give a concentration 0.1 mg/mL. Ten millilitres of the bioflocculant solution was heated at 50 °C, 60 °C, 70 °C and 80 °C for 30 min, and then the temperature dependence was determined by measuring the residual flocculating activity for kaolin suspension (4 g/L) at room temperature [14].

### 2.11. Fourier Transform Infrared Spectroscopy (FTIR)

The functional groups of the bioflocculant were determined using a Fourier transform infrared (FTIR) spectroscopy (Perkin Elmer System 2000, Cambridge, England). The bioflocculant was ground with KBr salt at 25 °C and pressed into a pellet for FTIR analysis over a wave number range of 4,000 to 350 cm^−1^ [19].

### 2.12. Thermo-Gravimetric Analysis (TGA)

Ten milligrams of the bioflocculant was analysed by TGA analyzer (STA 449/C Jupiter, Netzsch, Wittelsbacherstraße, Germany) over a temperature range of 20–900 °C with a heating rate of 10 °C per minute under a constant flow of nitrogen gas [14].

### 2.13. Chemical Composition of the Bioflocculant

The total protein content of the purified bioflocculant was determined as described by Bradford [20] using bovine serum albumin (BSA) as the standard solution. The total sugar content was determined by phenol-sulphuric acid method described by Chaplin and Kennedy [21] using glucose as a standard solution. The uronic acid content of the bioflocculant was determined by carbazole method described by Bitter and Muir [22].

### 2.14. Statistical Analysis

All data were treated in replicates and the mean values were taken. Data were subjected to one-way analysis of variance (ANOVA) using MINITAB Student Release 12 statistical package. A significance level of *p* < 0.05 was used.

## 3. Results and Discussion

### 3.1. Bioflocculant Yield

After fermenting 1 L of a mixed culture of *Halomonas* sp. Okoh and *Micrococcus* sp. Leo for 5 days, about 3.51 g of purified bioflocculant was recovered. Increase in the amount of bioflocculant recovered from the consortium compared to the individual strains (*Halomonas* sp. Okoh: 1.213 g/L; *Micrococcus* sp. Leo: 0.738 g/L) might be due to the synergistic effect observed when grown together. The higher bioflocculant yield observed with the consortium portends a reduction in production cost and as such, mixed culture fermentation may be preferred were the organisms act in synergy.

### 3.2. Time Course Assay of Bioflocculant Production

**Figure 1:**
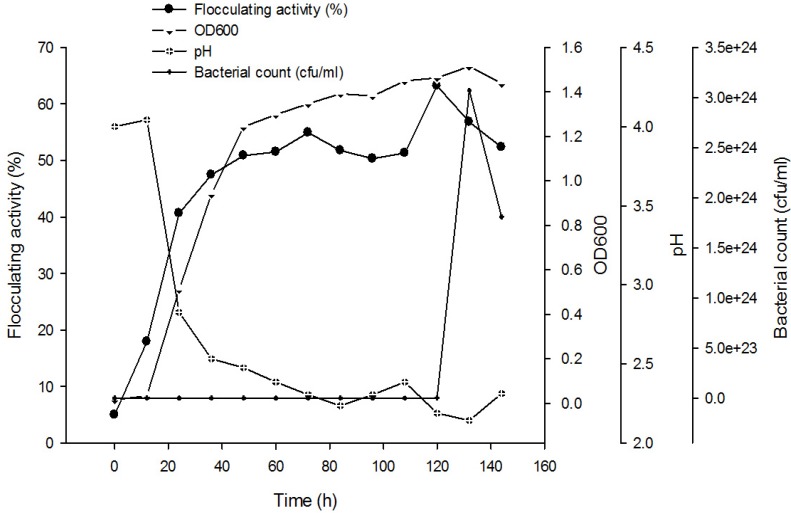
Time course for bioflocculant production by a mixed culture of *Halomonas* sp. Okoh and *Micrococcus* sp. Leo.

Optimum culture conditions that were used for bioflocculant production by individual strains (*Halomonas* sp. Okoh and *Micrococcus* sp. Leo) were adopted for culturing the consortium of the two strains. Figure 1 shows the time course assay of bioflocculant production. As expected, no cell growth was observed within the first 10 h of cultivation (lag phase). However, a steady increase in cell growth accompanied by a corresponding increase in flocculating activity was observed after this period. The stationary growth phase was attained after 120 h of cultivation. It was also observed that the flocculating activity ran parallel to cell growth, thus indicating a concomitant increase in bioflocculant production with cell growth. The flocculating activity of the bioflocculant reached its maximum flocculating peak of 63.2% at late stationary phase of 120 h and further increase in cultivation period resulted in a decrease in both flocculating activity and cell growth. This observation indicated that the production of bioflocculant was as a result of biosynthesis during the bacterial growth and not by cell autolysis [23]. The decrease in flocculating activity observed after 120 h could be attributed to the presence of bioflocculant-degrading enzymes produced by the microorganisms [4]. A similar observation was reported by Mabinya *et al.* [24] for the bioflocculant produced by *Halomonas* sp. Okoh which attained its maximum peak at 132 h. On the contrary, the flocculating activity of the bioflocculant produced by *Serratia fiacaria* and *Bacillus* sp. F19 reached its maximal at early stationary phase of 72 h [25,26]. The initial pH of the production medium was adjusted to 4 and then monitored at the regular intervals over the entire growth period. According to Salehizadeh and Shojaosadati [6], the pH of the production medium determines the electric charge of the cells and oxidation-reduction potential thus affecting nutrient absorption and enzymatic reactions. It was observed that there was a decrease in pH of the medium as cultivation time progresses. The decrease in pH of the medium may be due to the production of organic acids as a result of glucose metabolism since glucose was a component of the cultivation medium or the decrease in pH might be due to the presence of organic acids produced during metabolism by bacteria [7,27]. 

### 3.3. Effect of Bioflocculant Dosage on Flocculating Activity of Purified Bioflocculant

**Figure 2:**
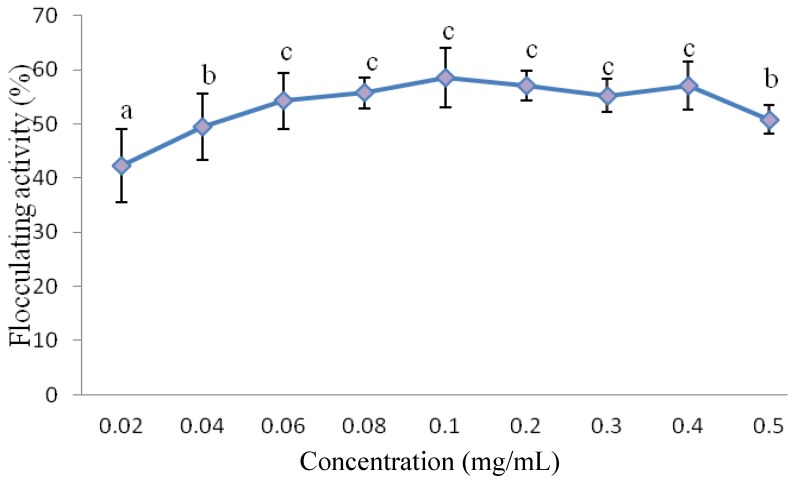
Effect of bioflocculant concentration on the flocculating activity of the bioflocculant produced by a consortium (*Halomonas* sp. Okoh and *Micrococcus* sp. Leo). Flocculating activities with different letters are significantly different (*p* < 0.05) from each other.

The appropriate bioflocculant concentration to be used for subsequent experiments was determined by investigating different bioflocculant concentrations ranging from 0.02–0.5 mg/mL as depicted in Figure 2. It was observed that the flocculating activity of the bioflocculant increased as the concentration increased. The optimum flocculating activity was obtained at 0.1 mg/mL and further increases in bioflocculant concentration resulted in a decline in the flocculating activity. According to the observation of Zufarzaana *et al.* [28], low dosage will not make bridging flocculation mechanism of the bioflocculant to be effective and high dosage will generate high viscosity which will inhibit the settling of suspended particles by restabilization of kaolin particles. When the bioflocculant molecules are excessively presence in the solution, they usually generate high viscosity, blocked the adsorption sites thereby reducing flocculating processing and flocs formation [14,25,28]. A similar observation was observed by Deng *et al.* [7] of the bioflocculant produced by *Bacillus mucilaginosus* that required a dosage of 0.1 mg/mL bioflocculant for optimum flocculating activity*.* On the contrary, the compound bioflocculant CBF-F26 produced by a mixed culture of *Rhizobium radiobacter* F2 and *Bacillus sphaeicus* F6 required a dosage of 12 mg/L for effective flocculating activity [14].

### 3.4. Thermostability of the Purified Bioflocculant

The relationship between temperature and flocculating activity of the bioflocculant was investigated (figure not shown). After heating 0.1 mg/mL solution of the purified bioflocculant at different temperatures ranging from 50–80 °C for 30 min, flocculating activity of the residual bioflocculant was measured at room temperature. There was a decrease in flocculating activity from 77.7% at 50 °C to approximately 70% at 80 °C. The bioflocculant maintained and retained about 70% of its flocculating activity at 80 °C due to its structure which is mainly composed of polysaccharide. Li *et al.* [29] reported a decrease of only 9.2% in flocculating activity of the bioflocculant produced by *Aeromonas* sp. after being heated at 100 °C for 60 min. Gong *et al.* [25] observed that the bioflocculant produced by *Serratia ficaria* could retain its flocculating activity after being heated at 100 °C for 15 min, mainly due to polysaccharide backbone. Li *et al.* [30] reported that the bioflocculant produced by *Agrobacterium* sp. M503 retained its flocculating activity up to 70 °C and a further increase in temperature up to 121 °C had no effect on flocculating activity. High thermostability property of a compound bioflocculant CBF-F26 was observed when the purified bioflocculant was heated over 100 °C for 30 min. The residual flocculating activity of this bioflocculant was more than 90% [14]. 

### 3.5. Effect of Cations on the Flocculating Activity of Purified Bioflocculant

**Figure 3:**
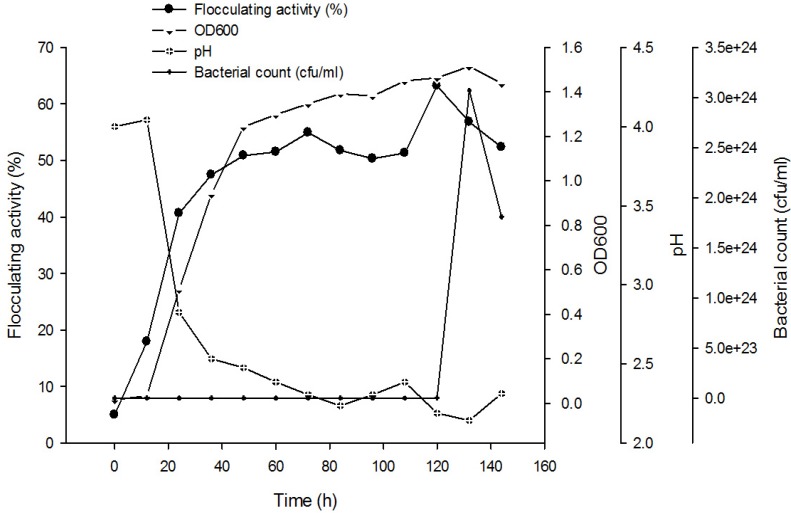
Effect of cations on the flocculating activity of the bioflocculant produced by a consortium (*Halomonas* sp. Okoh and *Micrococcus* sp. Leo). Flocculating activities with different letters are significantly different (*p* < 0.05) from each other.

The role of cations on the flocculating activity of the bioflocculant produced by the consortium was investigated and the results are depicted in Figure 3. Of all the cations tested, the flocculating activity of the bioflocculant was enhanced albeit to varying degrees by Ca^2+^ (72%), Mn^2+^ (59.8%) and Al^3+^ (80%) and inhibited to different extents by Li^+^ (12.2%), Na^+^ (18.8%), K^+^ (7.4%), Mg^2+^ (31.5%), Ba^2+^ (43.6%) and Fe^3+^ (36%). The role of cation is to neutralize and stabilize the negative charge of both functional groups of kaolin particle in solution and the bioflocculant [31,32]. Levy *et al.* [33] stated that the role of bivalent and trivalent cations is to increase the adsorption of bioflocculants on the suspended particles by decreasing the negative charge on both the polymer and the particle. Li *et al.* [4,34] also reported enhancement of flocculating activity of a bioflocculant produced by *Bacillus licheniformis* and *Bacillus circulans* in the presence of Al^3+^, Fe^3+^, and Ca^2+^. According to the investigation of Patil *et al.* [35] about the bioflocculant produced by a *Bacillus substilis,* the flocculating activity was stimulated in the presence of Al^3+^ and Fe^3+^. The compound bioflocculant produced by a mixed culture of *Rhizobium radiobacter* F2 and *Bacillus sphaeicus* F6, displayed flocculating activity of 97% when Al^3+^ was used as a coagulating aid [14]. 

On the contrary, Cosa *et al.* [36] reported that the flocculating activity of the bioflocculant produced by *Virgibacillus* sp. Rob was greatly stimulated when Fe^2+^ was used as the cation. The bioflocculant produced by *Chryserbacterium daeguense* and *Bacillus* sp. F19 required no cation for its flocculation efficiency [26,37].

### 3.6. Effect of pH on the Flocculating Activity of Purified Bioflocculant

**Figure 4:**
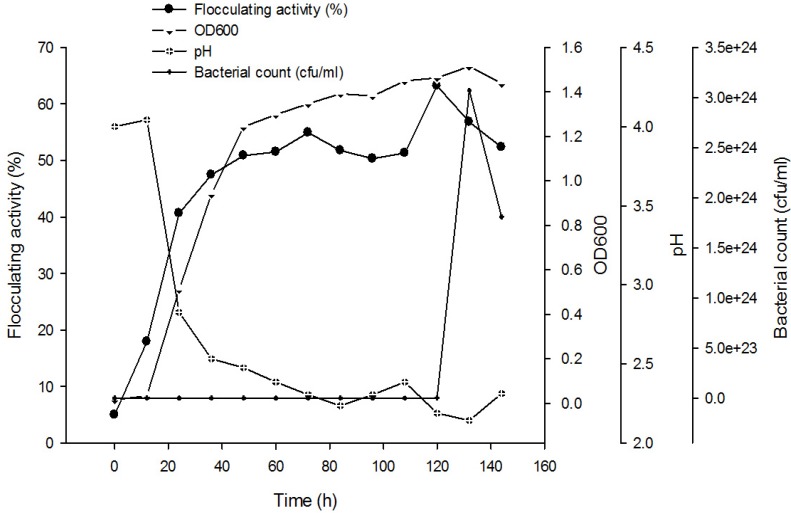
Effect of pH on the flocculating activity of the bioflocculant produced by a consortium (*Halomonas* sp. Okoh and *Micrococcus* sp. Leo). Flocculating activities with different letters are significantly different (*p* < 0.05) from each other.

The effect of pH on the flocculating activity of the compound bioflocculant was investigated using bioflocculant dosage of 0.1 mg/mL at different pH values ranging from 2–11. Figure 4 shows the results of the effect of pH on flocculating activity of the purified bioflocculant. It was observed that an increase in pH resulted into an increase in the flocculating activity of the produced bioflocculant. A sharp decreased in flocculating activity was recorded at pH 7 with an immediate rise up to pH 8 followed by a slight decrease and relative stability in pH. It was observed that the bioflocculant produced flocculated well at a wide pH range of 2–10 with the maximum flocculating activity peak of 86% at pH 8. Yokoi *et al.* [38] stated that the pH of the solution plays an important role in flocculating efficiency of the bioflocculant. Wang *et al.* [14] stated that the pH affected stability of suspended particles and the formation of flocs. Yim *et al.* [39] reported that the bioflocculant p-KG03 produced by a marine dinoflagellate, *Gyrodinium impudicum* KG03 flocculated best under acidic conditions of pH 4. The optimum pH for flocculating activity of the biopolymer produced by *Enterobacter cloacae* WD7 was 6 [40]. The compound biopolymer CBF-F26 produced by a mixed culture of *Rhizobium radiobacter* F2 and *Bacillus sphaeicus* F6 had flocculating activity between pH 7–9 [14].

### 3.7. Chemical Composition of the Purified Bioflocculant

Chemical analysis showed that the purified bioflocculant was composed of 4.73% total protein content, 62.3% total sugar content and 25.7% uronic acid. Wang *et al.* [14] reported the purified bioflocculant CBF-F26 mainly composed of polysaccharide with monosaccharide units of rhamnose, mannose, glucose and galactose respectively in a 1.3:2.1:10.0:1.0 molar ratios. 

### 3.8. Thermogravimetric Property of the Purified Bioflocculant

**Figure 5:**
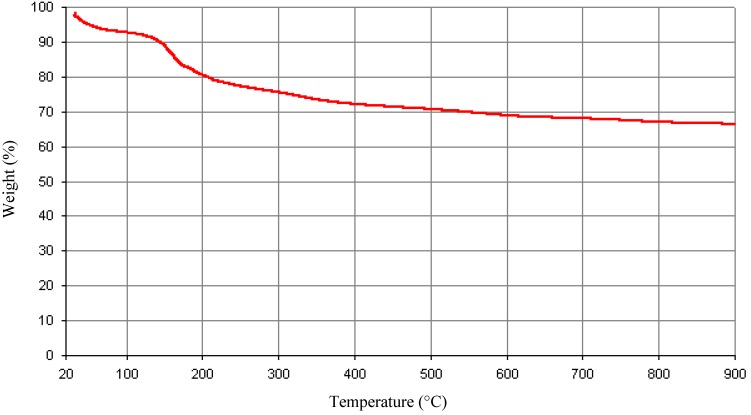
Thermogravimetric analyses of the purified bioflocculant.

The thermogravimetric property analysis of the purified bioflocculant was used to elucidate its behaviours when subjected to heat. This enables us to understand its pyrolysis property when exposed to a very high temperature. From Figure 5, there was about 20% decrease in weight at 200 °C and about 29% loss of weight at 500 °C. The first weight loss could be due to loss of moisture content in the bioflocculant [41]. Similarly, in the case of bioflocculant p-KG03 produced by a marine dinoflagellate *Gyrodinium impudicum* KG03 [39], the initial weight loss was observed between 40–230 °C. Further decrease in weight loss of this bioflocculant was observed at about 310 °C. Wang *et al.* [14] reported on a study conducted on a compound bioflocculant by a mixed culture of *Rhizobium radiobacter* F2 and *Bacillus sphaeicus* F6 where initial loss of about 10% was observed between 20 and 150 °C. Further decreased in weight of 40% was observed at 400 °C and there was a total loss of weight at 1,000 °C.

### 3.9. Functional Groups Determination by FTIR

**Figure 6:**
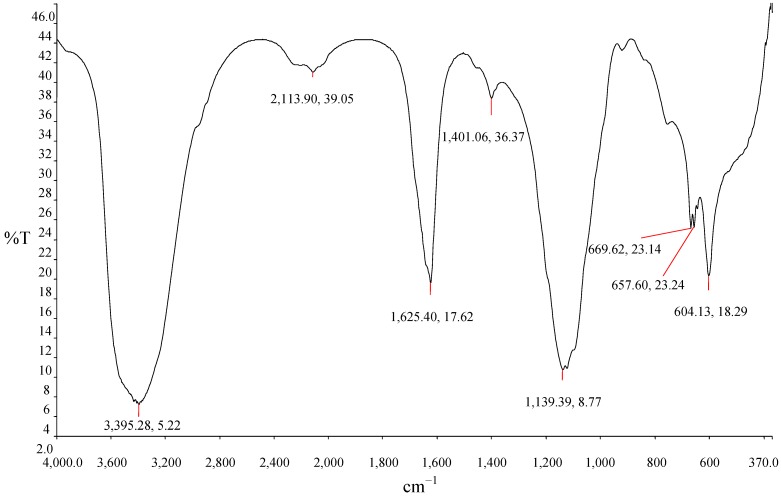
Fourier transform infrared (FTIR) spectroscopy of the purified bioflocculant.

The composition of the bioflocculants produced by different microorganisms differ [6]. The flocculating activity of the purified bioflocculant solely depends on the chemical structure which is related to the functional groups in the molecule. The Fourier-transform infrared (FTIR) spectrum analysis revealed the presence of different functional groups in the molecule. In Figure 6, the spectrum peak at 3,412 cm^−1^ showed the presence of OH group and NH_2_ group in the molecule [42]. The weak band noticed at 2,113 cm^−1^ indicated the presence of aliphatic bonds. The spectrum peak at 1,622 cm^−1^ is an indication of the presence of an amide group [9]. The vibration peak at 1,139 cm^−1^ corresponding to the C-O stretching in alcohols and this further suggests the presence of OH group in the bioflocculant molecule [43]. The spectrum peaks in between 1,000–1,100 cm^−1^ suggested the presence of saccharide derivatives.

## 4. Conclusions

The flocculating efficiency and physicochemical properties of the compound bioflocculant produced by a mixed culture of *Halomonas* sp. Okoh and *Micrococcus* sp. Leo were investigated. The bioflocculant maintained wide pH range flocculating activity with a maximum peak of 86% at pH 8. The glycoprotein bioflocculant possessed hydroxyl, carboxyl and amino groups in its molecule as the main functional groups which were responsible for the flocculation mechanism. Nonetheless, the high flocculation activity observed indicates prospects towards industrial applications, and in addition, further studies on process conditions are needed for the prospect of large-scale production.
